# Mpox Vaccine Hesitancy Among Brazilian Men Who Have Sex with Men: A National Cross-Sectional Study

**DOI:** 10.3390/vaccines12111229

**Published:** 2024-10-29

**Authors:** Guilherme Reis de Santana Santos, Caíque Jordan Nunes Ribeiro, José Flávio Cerqueira dos Santos Júnior, Valdemar Silva Almeida, Rita de Cassia Dias Nascimento, Nilo Manoel Pereira Vieira Barreto, Anderson Reis de Sousa, Márcio Bezerra-Santos, Lariane Angel Cepas, Ana Paula Morais Fernandes, Isabel Amélia Costa Mendes, Aires Garcia dos Santos Júnior, Maria Luisa Pereira Maronesi, Álvaro Francisco Lopes de Sousa

**Affiliations:** 1 Department of Nursing, Federal University of Sergipe, Lagarto 49400-000, SE, Brazil; guilhermereis22@academico.ufs.br (G.R.d.S.S.); caiquejordan@academico.ufs.br (C.J.N.R.); cerq.flavio@academico.ufs.br (J.F.C.d.S.J.); svaldemar687@academico.ufs.br (V.S.A.); 2 Graduate Program in Nursing, Federal University of Sergipe, São Cristóvão 49100-000, SE, Brazil; 3 School of Nursing, Federal University of Bahia, Salvador 40170-115, BA, Brazil; rita.nascimento@ufba.br (R.d.C.D.N.); nilo.manoel@ufba.br (N.M.P.V.B.); anderson.sousa@ufba.br (A.R.d.S.); 4 Complex of Medical Sciences and Nursing, Federal University of Alagoas, Arapiraca 57309-005, AL, Brazil; marciobezerra@arapiraca.ufal.br; 5 Ribeirão Preto College of Nursing, University of São Paulo, Ribeirão Preto 14040-903, SP, Brazil; larianeangelcepas@usp.br (L.A.C.); anapaula@eerp.usp.br (A.P.M.F.); iamendes@usp.br (I.A.C.M.); 6 NOVA National School of Public Health, Public Health Research Centre, Comprehensive Health Research Center, CHRC, REAL, NOVA University Lisbon, 1099-085 Lisbon, Portugal; 7 Postgraduate Program in Nursing, Federal University of Mato Grosso do Sul, Três Lagoas 79613-000, MS, Brazil; aires.junior@ufms.br (A.G.d.S.J.); maria.luisa.maronesi@ufms.br (M.L.P.M.); 8 Institute of Teaching and Research, Hospital Sírio-Libanês, São Paulo 01308-050, SP, Brazil

**Keywords:** mpox, vaccine, hesitancy, men who have sex with men, stigma, discrimination, sexual behavior, risk factors, vaccination acceptance

## Abstract

Background: Mpox is a viral zoonosis that has gained increased attention due to a global outbreak in 2022, significantly impacting men who have sex with men (MSM). Vaccination for this disease poses a public health challenge; because it carries a strong stigma, there may be greater hesitancy in vulnerable groups. Objectives: This study aimed to determine the prevalence and factors associated with Mpox vaccine hesitancy among Brazilian MSM. Methods: A cross-sectional study was conducted between September and December 2022 using an online survey targeted at MSM. Recruitment was carried out through social media and dating apps. The sample consisted of 1449 participants and the analysis involved bivariate logistic regression. Results: The prevalence of Mpox vaccine hesitancy was 7.57%. The significant factors associated with hesitancy were primarily related to sexual practices and attitudes towards Mpox exposure and diagnosis, such as not using “glory holes” (aOR: 19.82; 95% CI: 1.60–245.69), reluctance to undergo pre- and post-exposure testing for Mpox (aOR: 9.54; 95% CI: 5.52–16.48), and not knowing close contacts diagnosed with Mpox (aOR: 4.09; 95% CI: 1.72–9.73). Participants who would not take precautions after diagnosis (aOR: 3.00; 95% CI: 1.27–7.07) and those who would not disclose their serological status (aOR: 1.93; 95% CI: 1.13–3.30) also showed a higher likelihood of vaccine hesitancy. Conclusion: Public health strategies should address these factors to expand knowledge about vaccination barriers, plan educational campaigns with targeted messaging for the MSM population, and provide inclusive healthcare environments to increase vaccine acceptance and reduce Mpox transmission in vulnerable groups.

## 1. Introduction

The virus responsible for Mpox (MPXV) is a double-stranded DNA infectious agent belonging to the same genus as human smallpox, Orthopoxvirus, of the Poxviridae family, and can infect both humans and animals. With an incubation period of approximately one to two weeks, symptoms may include fever, body aches, lethargy, lymphadenopathy, and skin rashes. Sue to its similarity to smallpox, as they share the same viral genus, the vaccine used for smallpox eradication in the 1970s also provides immunity against Mpox. However, the absence of continuous vaccination campaigns after that period may have contributed to the global spread of Mpox [1,2,3].

Although it was first identified in 1958 in Denmark among monkeys used for research purposes, it gained greater recognition after reports of human cases in the endemic regions of West and Central Africa [4,5], particularly in what is now the Democratic Republic of the Congo, in 1970. Since then, the disease has remained confined in rural areas near tropical forests, where there is a higher likelihood of human interaction with wild, infected animals [6,7].

Historically, the primary modes of Mpox transmission to humans included bites or scratches from animals, the consumption of contaminated bushmeat, or close contact with the bodily fluids or skin lesions of an infected animal. Although less common, human-to-human transmission can occur through prolonged intimate contact, including sexual contact, direct contact with infectious lesions or bodily fluids, and large respiratory droplets. The transmission pattern has shown an increasing potential for intercontinental spread, associated with urbanization and increased human mobility [8,9].

The Mpox outbreak in 2022 rapidly spread across several previously non-endemic countries, causing a worldwide state of alert. During this period of global virus dissemination, a notable increase in incidence among men who have sex with men (MSM) was observed, indicating a shift in the conventional epidemiological pattern of the disease and suggesting a new possible route of transmission, facilitated by the close and prolonged contact typical of sexual encounters [10,11]. Additionally, large events with gatherings, such as parties and festivals, where there is a high concentration of people, especially MSM, also played an important role in the spread of the virus, contributing to the amplification of cases during the outbreak [12]. In Brazil, more than half of the confirmed cases were reported in São Paulo in 2024, while states like Roraima, Amapá, Tocantins, Maranhão, and Piauí did not register any suspected or confirmed cases [13].

Beyond concerns related to sexual behavior as a risk factor, the spread of Mpox among MSM during the 2022 outbreak also revisited issues of stigma and discrimination, which may exacerbate public health responses [11,14]. Research suggests that social gatherings, such as parties and sauna meetings typical among MSM, played a role in virus transmission. The stigma associated with this disease may also have impacted people’s willingness to get vaccinated and their readiness to seek medical care [15].

In 2023, Brazil implemented vaccination strategies targeting the most vulnerable groups at higher risk of exposure to the virus. According to the Ministry of Health, priority groups included people living with human immunodeficiency virus (HIV) or acquired immune deficiency syndrome (AIDS), healthcare workers involved in Mpox diagnosis, and individuals who had contact with bodily fluids from suspected, probable, or confirmed cases [16]. Reports indicated that vaccines were available, but access was limited to priority groups such as healthcare workers, MSM, and individuals who had direct contact with confirmed cases [17]. The distribution and availability of vaccines were hampered by a limited supply and the need to import doses, which hindered large-scale vaccination coverage. Additionally, the lack of public awareness also contributed to a limited perception of the importance of Mpox vaccination, affecting disease control [18].

In 2024, the World Health Organization (WHO) reclassified Mpox as a public health emergency of international concern due to the resurgence of cases. The threat is recurring, and there is once again the risk of a pandemic due to the rapid increase in cases, which are predominantly concentrated among MSM. Since the newly discovered strain has proven to be more virulent and possesses greater transmission potential, it is crucial to develop and implement immunization strategies to halt its spread [19].

Between January 2022 and June 2024, 99,176 confirmed cases of Mpox and 208 deaths were recorded in 116 countries, according to WHO data. During the same period, Brazil ranked as the second country with the highest number of confirmed cases globally, with 11,212 Mpox diagnoses. As of September 2024, 1015 confirmed or suspected cases were recorded in Brazil, which represents a higher number than in all of 2023 in the country. Additionally, men aged 18 to 39 years represented more than 70% of confirmed cases [20,21,22].

Although vaccination has become essential for Mpox control, several obstacles to its acceptance remain. Vaccine hesitancy among MSM has proven to be a significant barrier in Brazil [18,23]. The belief that the disease affects only a specific group leads to an underestimation of the need for the vaccine. Moreover, many people choose not to get vaccinated due to the stigma associated with the disease and fear of discrimination, contributing to the continued spread of the virus [24].

Vaccine hesitancy has serious repercussions, particularly for disadvantaged populations. The lack of adherence to vaccination and the underestimation of the vaccine can prolong outbreaks and increase the likelihood of viral mutations, which could reduce the efficacy of available vaccines. As a result, inadequate immunization may extend epidemics and increase the morbidity and mortality associated with Mpox [25,26]. Therefore, it is crucial to implement effective communication strategies to combat stigma, demystify the disease, and advocate for vaccination as a vital public health intervention, as well as to understand the characteristics of those who choose not to be immunized against Mpox.

This study is crucial due to the ongoing rise in confirmed cases in Brazil and the shift in Mpox virus’s epidemiological and transmission profiles, which has predominantly affected MSM. Understanding vaccine acceptance for Mpox is crucial for guiding public policy actions in the country and understanding the key behaviors of this group.

This study aimed to determine the prevalence and factors associated with Mpox vaccine hesitancy among Brazilian MSM during the 2022 outbreak.

## 2. Materials and Methods

### 2.1. Study Design

We conducted a cross-sectional, analytical, and quantitative study using an electronic survey. Data collection took place from September to December 2022, during which Brazil was experiencing an Mpox outbreak, with a rising number of cases predominantly in São Paulo and Rio de Janeiro.

### 2.2. Study Location

Data collection occurred in a virtual environment in Brazil during the latter half of 2022. The study utilized digital social networks such as Facebook^®^, Instagram^®^, and X^®^, along with messaging apps like WhatsApp^®^ and Telegram^®^. Additionally, social networks specifically popular among MSM, including Hornet^®^, Grindr^®^, Scruff^®^, and Tinder^®^, were used. These platforms, known for facilitating romantic and sexual encounters, offer a queer or gay-friendly perspective and are widely available in Brazil. A research-specific profile, which included a description of the study and proof of approval by the Research Ethics Committee (REC), was used to access these networks.

### 2.3. Population, Sample, and Inclusion Criteria

The study population comprised Brazilian men who used social networks and interaction apps. The inclusion criteria included identifying as male [either cisgender (individuals who identify with the gender assigned at birth) or transgender (individuals who do not identify with the gender assigned at birth)], having had at least one sexual encounter with another man in the past 12 months, being 18 years or older, and residing in Brazil. The exclusion criteria applied to men who, although residents of Brazil, were traveling internationally during the data collection period.

A sample size calculation for proportions was conducted using G*Power software (version 3.1.9.7), considering the population of men over 18 years old in Brazil (approximately 98.5 million men) [27]. An assumed prevalence of 50% was utilized (to maximize the sample size, given the lack of prior data on the prevalence of this phenomenon), with a tolerable standard error of 3% and a confidence level of 95%. This resulted in a required final sample size of at least 1067 men.

### 2.4. Data Collection Procedures

To achieve the desired sample size, we combined online recruitment strategies to select study participants. The first approach, adapted from previous studies, utilized the snowball sampling technique in the virtual environment [28,29,30,31]. Through this method, participants were encouraged to recruit individuals from their social circles using their online social networks and contacts. These individuals served as “seeds” for the study. Initially, 20 MSM were selected, representing diverse characteristics such as different Brazilian regions, self-reported races or skin colors (white or non-white), incomes, and education levels (elementary, high school, or higher education).

To identify the seeds, two trained and experienced researchers created public profiles on geolocation-based dating apps (Grindr^®^ and Hornet^®^). Through direct chat conversations with online users, each participant received a survey link accompanied by instructions to invite other MSM from their social networks, optimizing the sample size. This dissemination strategy engaged the first available individuals on both applications who met the inclusion criteria, aligning with recommendations from previous studies [29,30,31]. Simultaneously, this study was promoted using a secondary recruitment strategy on two widely used social media platforms: Facebook^®^ and Instagram^®^. This approach provided alternative avenues to connect with individuals beyond metropolitan areas, especially considering Brazil’s vast geographical landscape [29,30,31,32].

### 2.5. Collection Instrument

Data collection was conducted using the REDCap 12.0.0 platform (Vanderbilt University, Nashville, TN, USA), utilizing the software’s settings to prevent duplicate responses from the same participant. The form was validated through face-content validity (Content Validation Index: 0.96) by five experts and five participants. The survey consisted of four sections with a total of 46 questions, most of which were in a multiple-choice format. The questions covered various aspects, including social and demographic information (gender identity, sexual orientation, age, education level), sexual–affective relationships (type of partners, type of relationships, number of partners), sexual behaviors and practices, experience and proximity to Mpox, use of health services, and fear or stigma associated with Mpox.

### 2.6. Variables

(1) Dependent variable: Dichotomous outcome (yes or no) for the intention to get vaccinated against Mpox, if a vaccine were available.
(2) Independent variables:
  (a) Sociodemographic data: Variables analyzed included age, gender identity, sexual orientation, marital status, race/skin color, education level, formal income, and COVID-19 vaccination status.
  (b) Sexual behaviors and practices: Variables included sexual partnerships (fixed and casual); having unprotected sex (bareback); participation in chemsex, cruising, and orgies; and the frequency of using saunas and apps to find sexual partners.
  (c) Beliefs and information about Mpox: Beliefs about Mpox impact, previous diagnosis, intimate contact with diagnosed individuals, and fear of discrimination by healthcare services, friends, or family were included.

The following practices were defined based on previous studies [5,32,33]:

(a) Double penetration (DP): simultaneous sexual penetration by two or more penises.
(b) Challenging sexual practice: consistent engagement in two or three of these practices, defined by the circumstances in which they occur.
(c) Fisting or footing: anal penetration using the fist or foot.
(d) Cruising: free, consensual, and anonymous sex practiced between men in public spaces such as parks, forests, beaches, or parking lots.
(e) Glory hole: a round opening in a wall that allows the insertion of the penis, so someone on the other side can engage with it sexually in various ways.
(f) Bugchasing: when an HIV-negative man deliberately seeks out an HIV-positive man to become infected [34].

To define “chemsex”, participants were asked whether they had consumed illicit drugs immediately before and/or during sexual intercourse in the past 12 months. For those who responded affirmatively, a multiple-choice list was provided to indicate the specific drugs consumed [28].

### 2.7. Data Analysis

Statistical analyses were conducted using IBM SPSS 27.0 software (SPSS Inc., Chicago, IL, USA). An initial exploratory analysis was performed to describe the distribution of predictor variables according to vaccine hesitancy among study participants. The data were presented as absolute and relative frequencies.

Pearson’s Chi-square test was used to assess the association between predictor variables and vaccine hesitancy and select variables for inclusion in the multivariate model. A *p*-value of <0.20 was adopted as the statistical criterion for inclusion.

Multicollinearity was assessed using tolerance coefficients and variance inflation factor (VIF) parameters. The final stage of the analysis involved multivariate modeling to identify factors independently associated with vaccine hesitancy among MSM. A binomial logistic regression model was chosen, as the dependent variable was dichotomous, and the odds ratio (OR) was used because the prevalence of the outcome was <10%.

Adjusted odds ratios (aORs) and their respective 95% confidence intervals (95% CIs) were calculated to determine the strength of association between predictive variables and the outcome. The omnibus test was used to evaluate whether the final multivariate model better explained the factors associated with vaccine hesitancy compared to a model that included only the intercept. Statistical significance was set at 5% (*p*-value < 0.05). The Akaike Information Criterion (AIC), deviance, and log-likelihood parameters were used as model selection criteria, with lower values indicating a better fit. The significance of the aOR for variables in the final model was tested using the Wald Chi-square test, and variables with a *p*-value < 0.05 in the final model were considered significant.

### 2.8. Ethical Considerations

This study followed the ethical guidelines for research involving human subjects as outlined in the Declaration of Helsinki and Resolutions 466/2012 and 510/2016 of the Brazilian National Health Council. The research protocol was approved by the Ethics Committee of the School of Nursing at the Federal University of Bahia (UFBA) (CAAE: 61180222.8.0000.5531), and an Informed Consent Form (ICF) was presented to all invited participants.

## 3. Results

The final study sample consisted of 1449 MSM, with a vaccine hesitancy prevalence of 7.57% (*n* = 102). Table 1 presents the characteristics of the study participants, who were predominantly over 30 years of age (54.87%; 95% CI: 52.29–57.41), cisgender (96.62%; 95% CI: 95.56–97.43), gay (84.33%; 95% CI: 82.37–86.11), single (75.50%; 95% CI: 73.22–77.65), non-white (59.83%; 95% CI: 57.29–62.33), belonging to a religion (49.48%; 95% CI: 46.91–52.05), with higher education (88.06%; 95% CI: 86.29–89.63), and having a formal income (73.22%; 95% CI: 70.88–75.44).

Regarding sexual practices, the majority had both steady and casual partners (67.01%; 95% CI: 64.55–69.38), stopped having casual sex after the emergence of Mpox (55.14%; 95% CI: 52.57–57.69), practiced bareback (55.76%; 95% CI: 53.19–58.30), and used apps to search for sex (56.66%; 95% CI: 54.09–59.19). Additionally, most participants did not use saunas for sexual encounters (85.02%; 95% CI: 83.09–86.77), did not seek sex in public places such as parties and clubs (68.74%; 95% CI: 66.30–71.07), and were not involved in chemsex (80.54%; 95% CI: 78.42–82.49). On the other hand, smaller proportions reported engaging in challenging sexual practices (15.46%; 95% CI: 13.69–17.41), practicing cruising (13.04%; 95% CI: 11.41–14.88), participating in orgies (11.11%; 95% CI: 9.59–12.83), practicing bugchasing (8.60%; 95% CI: 7.35–10.26), and using glory holes (6.55%; 95% CI: 5.39–7.95).

Regarding variables related to Mpox, the majority had not been previously diagnosed with Mpox (85.44%; 95% CI: 83.53–87.16), did not know anyone close who had been diagnosed with Mpox (74.53%; 95% CI: 72.23–76.71), had no close contact with people diagnosed (87.92%; 95% CI: 86.14–89.50), and stated that they would disclose their Mpox serological status (78.60%; 95% CI: 76.42–80.64). Furthermore, they reported fear of discrimination in health services (55.90%; 95% CI: 53.33–58.44), from friends (65.42%; 95% CI: 62.94–67.83), and from family (51.28%; 95% CI: 48.70–53.84). Additionally, they believed that people could suffer from homophobia due to Mpox (84.06%; 95% CI: 82.08–85.85) (Table 1).

The best-fit binomial logistic regression model identified six variables independently associated with higher odds of Mpox vaccine hesitancy among Brazilian MSM. Not using a glory hole was linked to almost 20 times higher odds of not getting vaccinated against Mpox (aOR: 19.82; 95% CI: 1.60–245.69). Health vulnerability behaviors, such as stating that they would not perform pre- and post-exposure testing if available (aOR: 9.54; 95% CI: 5.52–16.48), not seeking testing centers (aOR: 6.09; 95% CI: 2.62–14.13), not taking the necessary precautions if diagnosed with Mpox (aOR: 3.00; 95% CI: 1.27–7.07), and not disclosing their Mpox serological status (aOR: 1.93; 95% CI: 1.13–3.30) were also significantly associated with vaccine hesitancy. Additionally, not having close acquaintances diagnosed with Mpox was associated with a four times greater likelihood of vaccine hesitancy (Table 2).

## 4. Discussion

Vaccine hesitancy is a public health phenomenon influenced by misinformation, misguided beliefs, pseudoscientific narratives, temporal correlations with adverse effects, neglect of the severity of past epidemics, and skepticism towards health institutions [35,36]. In our data, we observed a low prevalence of vaccine hesitancy against Mpox among a representative sample of MSM in Brazil (7.57%). Similarly, in China, the prevalence of vaccine hesitancy for Mpox ranges from 5.59% [37] to 13.85% [38] within the same demographic. On the other hand, among those diagnosed with HIV, studies indicate that 56.8% of seropositive MSM in China [39] and 59.8% in France [40] were willing to receive the vaccine.

In the general population, willingness to use the Mpox vaccine varies from 29.0% in Romania to 81.5% in the Netherlands [41,42], demonstrating that values differ across various populations and regions [43]. These data emphasize the importance of examining the causes and specifics of this hesitancy in vulnerable and stigmatized groups that face barriers in accessing health services.

Since Mpox currently seems to spread through practices commonly associated with the MSM community [44], understanding the role of these actions in vaccine willingness is crucial. Practices such as using glory holes, participating in parties and social gatherings, using apps to seek sex, and other forms of intimate contact can influence both risk perception and adherence to preventive measures like vaccination [45,46]. Understanding how specific behaviors impact the decision to vaccinate can support the development of effective public health policies that consider the unique characteristics of vulnerable groups. Additionally, it can help dismantle prejudices and stigmas, promoting a more inclusive and receptive environment for vaccination, which is essential for containing the spread of Mpox [39,40].

One of the most significant findings was the association between avoiding the practice of glory holes and a higher risk of vaccine hesitancy. Specifically, the odds of not receiving the Mpox vaccine were nearly 20 times higher among those who did not engage in this practice (aOR: 19.82; 95% CI: 1.60–245.69). This practice, which involves sexual contact with minimal physical interaction beyond the sexual organ, has been identified as a preventive measure against infections, particularly during the COVID-19 pandemic [47]. It is possible that those who avoid this practice, perhaps due to its association with multiple partners in a short period, may underestimate the risk of contracting Mpox, leading them to believe that vaccination is unnecessary in their health context.

The rise of Mpox has sparked renewed discussions about prevention and care practices, as the sexual behavior of many MSM has been modified due to heightened awareness of transmission risks. In this study, 55.14% of participants reported ceasing casual sexual relations in response to the Mpox outbreak. Other studies have also reported a reduction in sexual activity following the emergence of Mpox, with declines ranging from 53.9% to 64.6% [45,48]. Conversely, Prochazka et al. (2024) [49] observed a 2.5-fold reduction in sexual partners after the disease emerged, but noted that participants who had already been vaccinated against Mpox or had contracted the disease were less likely to continue with behavioral adaptations.

A survey conducted among Brazilian cisgender men within the LGBTQIA+ community indicated that approximately 96.9% were aware of Mpox, and 95.1% were willing to be vaccinated [50]. However, only 62% of the vaccine doses made available to target audiences were utilized, as demonstrated by the Ministry of Health [17]. For instance, in a sample population from Rio de Janeiro that met the vaccination criteria for Mpox, only 43% had been immunized. Reasons such as lack of knowledge about vaccine locations and availability, belief that the vaccine was experimental, perceived low risk of infection, and concerns about adverse effects were linked to vaccine hesitancy [18].

Since July 2022, when the World Health Organization (WHO) declared Mpox a Public Health Emergency of International Concern, the Brazilian government has implemented several initiatives. These include expanding diagnostic capabilities by establishing 27 central laboratories and three national reference laboratories, immunizing those at higher risk for severe forms of the disease, conducting workshops on HIV/AIDS, promoting national webinars, and actively publishing and disseminating contingency plans, epidemiological bulletins, protocols, and informational notes [51]. Despite these efforts to increase public awareness, vaccine hesitancy among at-risk populations [18] and the persistence of misinformation [11] continue to challenge public health efforts in Brazil. It is important to note that this study was conducted before many of these government responses in 2022. Therefore, the results reflect a snapshot of that period, and subsequent policies and initiatives may have influenced the findings presented here.

The willingness to forego pre- and post-exposure testing for Mpox was identified as another susceptibility factor, significantly increasing the likelihood of vaccine hesitancy (aOR: 9.54; 95% CI: 5.52–16.48). Reluctance to participate in preventive health activities may stem from a lack of awareness of the risks involved, distrust of health services, or a desire to avoid the stigma associated with an STI diagnosis. A study conducted in China among university students found that a low level of knowledge about Mpox and inadequate risk perception were linked to increased vaccine hesitancy [52], emphasizing the need to establish and expand effective communication channels between the public and health authorities.

Higher vaccine hesitancy was also associated with reluctance to seek specialized testing facilities (aOR: 6.09; 95% CI: 2.62–14.13). This relationship may be due to MSM’s lack of access to welcoming and inclusive services or fear of prejudice or criticism from medical professionals. Testing and vaccination are more likely when individuals trust the healthcare system and view it as a safe and respectful environment [53,54]. Thus, the association between MSM and Mpox in Brazil may further hinder access to healthcare services for this population, adding to the vulnerabilities faced in accessing sexual health care by socially disadvantaged groups [50,55].

Not taking appropriate precautions in the case of an Mpox diagnosis (aOR: 3.00; 95% CI: 1.27–7.07) was also linked to vaccine hesitancy. This behavior could indicate ignorance or a misperception of the disease’s severity. Health education campaigns that highlight the harmful effects of Mpox and the importance of treatment and prevention could help counter these misconceptions and reduce vaccine hesitancy.

There was also a higher likelihood of vaccine hesitancy when individuals chose not to disclose their Mpox serological status (aOR: 1.93; 95% CI: 1.13–3.30). Fear of discrimination and social stigma may prevent people from sharing their status, which could, in turn, decrease the likelihood of vaccination. This underscores the need for policies that foster an inclusive and supportive environment while reducing the stigma associated with STIs.

Interestingly, vaccine hesitancy increased fourfold when there were no close contacts diagnosed with Mpox. This suggests that exposure to Mpox cases, either directly or indirectly, may elevate risk perception and, consequently, the inclination to get vaccinated. Educational campaigns, when well executed, have the potential to raise awareness about the importance of vaccination and improve adherence, particularly among individuals who perceive a higher risk [56]. The success of these campaigns often hinges on culturally appropriate messaging and community engagement. Another approach, using motivational interviewing techniques, appears effective in addressing individual hesitations and encouraging vaccination. Moreover, training local leaders through community engagement has proven to be an effective tool [57].

Despite efforts by academic and health networks, some population groups may remain resistant due to personal beliefs or sociopolitical stigmas surrounding vaccination [58,59].

Studies indicate that exposure to positive information increases vaccine acceptance [60,61,62]. However, there is also a direct effect after vaccination, where previously adopted preventive behaviors may relax and incomplete vaccination rates may rise, potentially compromising public health efforts to control the disease. Thus, our study results, along with the most recent literature on vaccine hesitancy, suggest that individual decisions regarding vaccines are shaped by perceptions of long-term risks and benefits. In the case of Mpox vaccine hesitancy among MSM, our data corroborate the existing literature showing that distrust in healthcare institutions and stigma play significant roles [60,61,62]. Therefore, public campaigns must not only combat stigma but also emphasize the importance of maintaining preventive behaviors, even after vaccination.

The results of this study underscore the importance of addressing the barriers to vaccination within specific communities, such as MSM, through targeted communication strategies, health education, and inclusive policies that build trust in medical services. To increase vaccination rates and safeguard public health, campaigns that highlight the benefits of immunization, reduce stigma, and provide accurate information about vaccine safety and efficacy are essential.

Consequently, this study not only advances our understanding of the factors influencing Mpox vaccine hesitancy among MSM in Brazil but also offers valuable insights for developing intervention strategies aimed at increasing vaccination rates and reducing Mpox transmission. The success of immunization campaigns and the protection of MSM communities’ health depend heavily on the implementation of comprehensive and culturally sensitive strategies.

## 5. Contributions to Public Health

Since the onset of the HIV/AIDS epidemic in the 1980s, the LGBTQIA+ population has faced the consequences of stigma in various aspects of their lives, including the association of their sexual behaviors with promiscuity. In this regard, the emergence of an infectious disease with a higher prevalence among men who have sex with men (MSM) in previously unaffected regions has exacerbated existing health inequalities and further fueled prejudice [63].

In this context, it is crucial to evaluate the barriers that vulnerable groups like MSM face when accessing healthcare facilities and services, particularly regarding preventive measures such as vaccination and the reasons for refusal. Understanding the key factors associated with vaccine hesitancy among MSM during public health emergencies can provide a foundation for the development of international public health interventions for this population and offer broader insights into MSM behaviors. Additionally, different groups have unique social determinants of health that influence their adherence to health practices and services, highlighting the importance of conducting studies on specific populations.

Government institutions should propose public health policies that directly address the vaccination barriers identified in this study. It is recommended that culturally sensitive public awareness campaigns be implemented, focusing on the MSM population, with clear messaging about the importance of the Mpox vaccine and its availability at healthcare services or partner non-governmental organizations. Furthermore, the establishment of community-based vaccination centers specializing in MSM care could increase trust in healthcare services. Specific training for healthcare professionals to better address the needs of this population without prejudice or stigma is also essential.

The availability of accessible rapid testing for Mpox that can be done at home, combined with an expansion of vaccination sites in high-concentration MSM areas, such as saunas and clubs, could facilitate access and reduce vaccine hesitancy. These efforts, along with campaigns involving community leaders and influencers from the LGBTQIA+ community, can ensure greater vaccine uptake and strengthen public health strategies.

## 6. Limitations

This study presents several limitations that should be considered when analyzing the results. First, due to the cross-sectional design, it is impossible to establish a causal relationship between the identified characteristics and vaccine hesitancy against Mpox. Additionally, there may be selection bias, as data were collected exclusively online through surveys on social media platforms and interaction apps, limiting the generalizability of the results to the entire MSM community in Brazil. The reliance on self-reported data for information about sexual practices, attitudes, and behaviors related to vaccination is another significant issue. Some participants may have withheld complete and accurate information due to stigma and fear of prejudice associated with their sexual activities and serological status.

Moreover, since this study was conducted over a limited time frame—between September and December 2022—it may not have captured changes in public opinion or behavior resulting from new knowledge about Mpox, vaccine availability, or awareness efforts initiated after this period. Finally, given the wide variety of communication channels used for data collection, there is a possibility that some participants may have been included more than once in the database, although the platform used for the research (REDCap) has data quality mechanisms to prevent this from happening [64].

Future research should focus on investigating vaccine hesitancy among Brazilian MSM beyond 2022, as many of the government responses to the disease occurred after that year. Additionally, the emergence of a new genetic clade of the virus in 2024 may further influence outcomes regarding the intention to vaccinate against Mpox in Brazil, in combination with the educational campaigns and measures implemented during this period.

## 7. Conclusions

A low level of vaccine hesitancy against Mpox was observed among Brazilian MSM who experienced the Mpox health crisis in 2022. However, several factors contributing to lower vaccination adherence raise important concerns about MSM behaviors in response to health threats during a public health emergency. It is clear that much of the challenge faced in Brazil stems from the lack of trust this group has in accessing healthcare services, as well as a lack of awareness about the disease’s severity. The association between not seeking Mpox testing services, not knowing close contacts diagnosed with the disease, and higher levels of vaccine hesitancy found in our study supports the hypothesis that there is a pressing need to expand public health policies aimed at disseminating reliable information. This also highlights the importance of adopting preventive measures and engaging in health-related behaviors.

## Figures and Tables

**Table 1:** Bivariate analysis of factors associated with vaccine hesitancy for Mpox among MSM in the context of Mpox transmission in Brazil, 2022.

Variables		Vaccine Hesitancy for Mpox				*p*-Value
		Yes		No		
		(n = 102)		(n = 1347)		
		n	%	n	%	
Sociodemographic data						
Young adult (<30 years)	Yes	41	6.3	613	93.7	0.305
	No	61	7.7	734	92.3	
Gender identity	Trans	2	4.1	47	95.9	0.575
	Cis	100	7.1	1300	92.9	
Homosexual orientation	Yes	87	7.1	1135	92.9	0.888
	No	15	6.6	212	93.4	
Marital status	Single	75	6.9	1019	93.1	0.633
	With a partner	27	7.6	326	92.4	
Race or skin color	Non-white	62	7.2	805	92.8	0.917
	White	40	6.9	542	93.1	
Religion	Yes	57	7.9	660	92.1	0.215
	No	44	6.1	672	93.9	
Higher education	Yes	86	6.7	1190	93.3	0.265
	No	16	9.2	157	90.8	
Formal income	Yes	71	6.7	990	93.3	0.417
	No	31	8.0	357	92.0	
Was immunized against COVID-19	Yes	102	7.0	1345	93.0	1.000
	No	0	0	2	100.0	
Sexual behaviors and practices						
Type of sexual partnership (last 6 months)	No partners	7	7.9	82	92.1	0.813
	Fixed	29	7.7	348	92.3	
	Fixed + casual	66	6.8	905	93.2	
Stopped casual sex after the Mpox outbreak	Yes	55	6.9	744	93.1	0.837
	No	47	7.2	603	92.8	
Bareback practice	Yes	43	5.3	765	94.7	0.005
	No	59	9.2	582	90.8	
Chemsex practice	Yes	27	9.6	255	90.4	0.070
	No	75	6.4	1092	93.6	
Cruising practice	Yes	13	6.9	176	93.1	1.000
	No	89	7.1	1171	92.9	
Challenging sexual practices	Yes	20	8.9	204	91.1	0.255
	No	82	6.7	1143	93.3	
Bugchasing practice	Yes	20	15.9	106	84.1	<0.001
	No	82	6.2	1241	93.8	
Participates in orgies	Yes	10	6.2	151	93.8	0.746
	No	92	7.1	1196	92.9	
Use of glory hole	Yes	2	2.1	93	97.9	0.059
	No	100	7.4	1254	92.6	
Use of apps to seek sex	Yes	54	6.6	767	93.4	0.469
	No	48	7.6	580	92.4	
Use of saunas to seek sex	Yes	7	3.2	210	96.8	0.020
	No	95	7.7	1137	92.3	
Seeking sex in public places (parties and clubs)	Yes	23	5.1	430	94.9	0.059
	No	79	7.9	917	92.1	
Beliefs and information about Mpox						
Previous Mpox diagnosis	Yes	19	9.0	192	91.0	0.243
	No	83	6.7	1155	93.3	
Knows close people who have been diagnosed with Mpox	Yes	14	3.8	355	96.2	0.006
	No	88	8.1	992	91.9	
Intimate contact with a person diagnosed with Mpox	Yes	19	10.9	156	89.1	0.041
	No	83	6.5	1191	93.5	
Would disclose Mpox serological status	Yes	72	6.3	1067	93.7	0.044
	No	30	9.7	278	90.3	
Fear of being discriminated against by healthcare services	Yes	55	6.8	755	93.2	0.679
	No	47	7.4	586	92.6	
Fear of being discriminated against by friends	Yes	65	6.9	883	93.1	0.746
	No	37	7.4	464	92.6	
Fear of being discriminated against by family	Yes	54	7.3	689	92.7	0.759
	No	48	6.8	658	93.2	
Belief that one might suffer homophobia because of Mpox	Yes	68	5.6	1150	94.4	<0.001
	No	34	14.8	195	85.2	
Would undergo pre- and post-exposure testing for Mpox	Yes	50	4.1	1166	95.9	<0.001
	No	33	30.3	76	69.7	
If there are specialized centers and/or testing services for Mpox, would you seek out one of these health services?	Yes	86	6.2	1310	93.8	<0.001
	No	16	30.2	37	69.8	
If you are diagnosed with Mpox, would you take steps to take care of yourself and inform the people you have been in contact with?	Yes	89	6.4	1298	93.6	<0.001
	No	13	21.0	49	79.0	
If you are diagnosed with Mpox, would you follow the treatment or recommendations of healthcare professionals?	Yes	98	6.9	1331	93.1	0.047
	No	4	20.0	16	80.0	

**Table 2:** Multivariate analysis of factors associated with vaccine hesitancy among MSM in the context of Mpox transmission in Brazil, 2022.

Variables	β	aOR	CI95%		*p*-Value	VIF	Tolerance
			Lower	Superior			
Not using a glory hole	2.99	19.82	1.60	245.69	0.020	1.11	0.90
Would not undergo pre- and post-exposure testing for Mpox	2.25	9.54	5.52	16.48	<0.001	1.02	0.98
Would not seek out specialized centers and testing services for Mpox	1.81	6.09	2.62	14.13	<0.001	1.07	0.94
Not knowing close people who have been diagnosed with Mpox	1.41	4.09	1.72	9.73	<0.001	1.01	0.99
Would not take steps to take care of yourself and communicate with people you have been in contact with if diagnosed with Mpox	1.10	3.00	1.27	7.07	0.012	1.08	0.93
Would not disclose Mpox serological status	0.66	1.93	1.13	3.30	0.001	1.03	0.97

Omnibus test [X^2^(6) = 125; *p*-value < 0.001]. Deviance: 495; AIC: 509; BIC: 545.

## Data Availability

Data connected to this research are available from the corresponding author upon request (AFLS).
